# Efficient Multiplex Genome Editing Induces Precise, and Self-Ligated Type Mutations in Tomato Plants

**DOI:** 10.3389/fpls.2018.00916

**Published:** 2018-07-03

**Authors:** Ryosuke Hashimoto, Risa Ueta, Chihiro Abe, Yuriko Osakabe, Keishi Osakabe

**Affiliations:** ^1^Graduate School of Advanced Technology and Science, Tokushima University, Tokushima, Japan; ^2^Graduate School of Technology, Industrial and Social Sciences, Tokushima University, Tokushima, Japan

**Keywords:** CRISPR/Cas9, multiplex genome editing, *SlEF1*α promoter, tomato, tRNA

## Abstract

Several expression systems for multiple guide RNA (gRNAs) have been developed in the CRISPR/Cas9 (clustered regularly interspaced short palindromic repeats/CRISPR associated protein 9) system to induce multiple-gene modifications in plants. Here, we evaluated mutation efficiencies in the tomato genome using multiplex CRISPR/Cas9 vectors consisting of various *Cas9* expression promoters with multiple gRNA expression combinations. In transgenic tomato calli induced with these vectors, mutation patterns varied depending on the promoters used to express *Cas9*. By using the tomato *ELONGATION FACTOR-1*α (*SlEF1*α) promoter to drive *Cas9*, occurrence of various types of mutations with high efficiency was detected in the tomato genome. Furthermore, sequence analysis showed that the majority of mutations using the multiplex system with the *SlEF1*α promoter corresponded to specific mutation pattern of deletions produced by self-ligation at two target sites of CRISPR/Cas9 with low mosaic mutations. These results suggest that optimizing the *Cas9* expression promoter used in CRISPR/Cas9-mediated mutation improves multiplex genome editing, and could be used effectively to disrupt functional domains precisely in the tomato genome.

## Introduction

Genome engineering has been used widely to perform functional gene modification in various organisms. The CRISPR/Cas9 system, consisting of Cas9 nuclease and a guide RNA (gRNA), is one of the most convenient genome editing tools currently available (Cong et al., 2013). Cas9 is an RNA-guided endonuclease that forms a Cas9/gRNA complex that can generate a double-strand break (DSB) at the target site(s). The DSB is then repaired, most frequently by non-homologous end joining (NHEJ), which can create insertion, deletion, and/or substitution mutations at the cleavage site, inducing disruption of gene function. Successful use of the CRISPR/Cas9 system has been reported in various plant species (Nishitani et al., 2016; Nomura et al., 2016; Liu et al., 2017), allowing the numerous advantages of this system to be applied to molecular genetic studies in various plants.

The CRISPR/Cas9 system is also useful for multiplex genome editing, in which modification of multiple loci can be performed simultaneously by multiple or single target-specific gRNA(s). Several gRNA expression systems for multiple site-directed mutagenesis have been reported; these include assembly of multiple and individual gRNA expression cassettes in a plasmid (Xing et al., 2014; Lowder et al., 2015; Ma et al., 2015) and co-expression of Cas9, gRNA, and a self-cleaving hammerhead ribozyme from a single Pol II promoter with subsequent cleavage to molecular units by the ribozyme (Tang et al., 2016). Production of multiple gRNAs as a single transcript followed by division into individual gRNAs by endonucleases post-transcriptionally has also been reported (Xie et al., 2015). In this latter system, the CRISPR-associated RNA endoribonuclease Csy4 (Čermák et al., 2017) and endogenous RNA processing enzymes are utilized to process pre-tRNA (Xie et al., 2015).

Although several gRNA expression systems for multiplex genome editing have been developed in the context of the CRISPR/Cas9 system, optimization of the promoter used to express *Cas9* has not yet been widely attempted in multiple site-directed mutagenesis in plant cells. CRISPR/Cas9-mediated genome editing using tissue-specific promoters for *Cas9* expression in egg cells, germ cells, or meristematic cells has been reported, and such optimization was shown improved mutagenesis efficiency in the Arabidopsis genome (Gao et al., 2015; Hyun et al., 2015; Wang et al., 2015; Yan et al., 2015; Mao et al., 2016; Osakabe et al., 2016; Tsutsui and Higashiyama, 2016; Osakabe and Osakabe, 2017). Thus, the use of tissue-specific promoters for *Cas9* expression could improve multiple mutagenesis in plant cells.

Here, we report an efficient system using a tissue-specific promoter for a *Cas9*- and tRNA-processing-based gRNA expression system in CRISPR/Cas9 editing, and its application to induce modification of multiple targets in tomato cells. We found that a combination of the tomato *ELONGATION FACTOR-1*α gene (*SlEF1*α) promoter to drive *Cas9* and the tRNA processing-based gRNA expression system greatly increased the efficiency of self-ligated type mutations in tomato. Using this system, a high mutation efficiency, combined with low mosaic mutations, was also detected at various target sites in the tomato genome. These results suggest that optimizing *Cas9* expression promoter for multiplex genome editing will further improve one of the most useful genome editing tools in plants.

## Materials and Methods

### Plant Material, Growth Conditions, and Transformation

*Solanum lycopersicum* L. cv. Micro-Tom was used in this study. Tomato plants were grown in a growth chamber under conditions of 21–25°C with 16 h light at 4000–6000 l×/8 h dark. CRISPR/Cas9 vectors were transformed into *Agrobacterium tumefaciens* strain GV2260 and introduced into tomato cotyledons by the leaf disk method according to a previous study with slight modification (Sun et al., 2006). Sterilized tomato seeds were germinated on MS medium and cotyledons (5–7 days after germination) were cut into small pieces of approximately 0.5–0.7 cm and then transformed with *Agrobacterium* (OD_600_ = 0.01) in 40 ml infection medium [1X MS liquid medium (pH5.7), 1.2 μl 2-mercaptoethanol (Sigma-Aldrich), 100 μM acetosyringone (TCI chemicals)]. The explants were transferred to MS medium containing 40 μM acetosyringone and cultured in the dark for 3 days in a growth chamber, then transferred onto MS-agar medium containing 100 mg/L kanamycin, 1.0 mg/L trans-zeatin (Wako), and 25 mg/L meropenem (Wako). Four weeks after transformation, transgenic calli were selected using kanamycin and GFP selection as a *Cas9* expression marker (Ueta et al., 2017). GFP positive calli were cut using a scalpel under a fluorescence stereoscopic microscope M165FC (Leica) for use in further mutation analysis.

### Plasmid Vector Construction and Design of tRNA–gRNA Units

The tRNA-based multiplex CRISPR/Cas9 vector was constructed according to Xie et al. (2015) with several modifications. The all-in-one plasmids were constructed based on pEgP237-2A-GFP (2 × *CaMV35S* Ω promoter) and pEgPubi4_237-2A-GFP (parsley *Ubiqutin4-2* promoter) (Ueta et al., 2017) as the vector backbone containing the tRNA sequences (5′-AACAAAGCACCAGTGGTCTAGTGGTAGAATAGTACCCTGCCACGGTACAGACCCGGGTTCGATTCCCGGCTGGTGCA-3′ and gRNA scaffold) and were named pMgP237-2A-GFP and pMgPubi4_237-2A-GFP, respectively. The *SlEF1*α promoter [the 1.5 kb upstream region of *SlEF1*α; *Solyc06g005060*, a homolog of Arabidopsis *ELONGATION FACTOR α-1* (At*EF1*α) (Osakabe et al., 2016)] and the tomato ribosomal protein *p16* promoter [the 1.0 kb upstream region of *Slp16*; *Solyc08g007140*, a homolog of Arabidopsis ribosomal protein *p16* (*Atp16*) (Forner et al., 2015)] were amplified by PCR from the tomato genome, and these promoter regions were replaced with the 2 × *CaMV35S* Ω in pMgP237-2A-GFP. These constructed vectors were named pMgPsef1_237-2A-GFP and pMgPs16_237-2A-GFP, respectively. The specific target sequences for the tRNA–gRNA units were selected using the web-tool “focas” (Osakabe et al., 2016) or Cas-OT software (Xiao et al., 2014). The units containing two gRNAs were amplified with primers Fw_tgRNA–gRNA and Rv_tgRNA–gRNA for each target (Supplementary Table S1) using the gRNA scaffold–tRNA template. The unit containing two gRNAs–tRNA was then inserted into the BsaI site of multiplex CRISPR/Cas9 vectors using Golden Gate Cloning methods.

The *SlEF1*α-GFP vector was constructed using the same promoter fragment from the multiplex vector and the pRI 35S-GFP (CaMV35S-GFP in this manuscript) (Yamada et al., 2016) as a backbone vector. *SlEF1*α promoter sequences were amplified from pMgPsef1_237-2A-GFP by PCR, and that fragment was inserted into the HindIII/XbaI sites of pRI 35S-GFP by the SLiCE reaction (Okegawa and Motohashi, 2015). All primers used in plasmid construction are listed in Supplementary Table S1.

### Mutation Analyses in CRISPR/Cas9 Target Sites

Genomic DNA was isolated from transgenic CRISPR/Cas9 tomato calli selected by observing high GFP fluorescence 4 weeks after transformation. To analyze the mutations or large deletions in the transgenic calli, the region including the target sites of gRNAs was amplified by PCR using PrimeSTAR GXL DNA Polymerase (TaKaRa) and analyzed by agarose-gel electrophoresis. In the Cel-1 assay, an amplified 300–500 bp region at the target locus was digested with Surveyor Mutation Detection Kits (IDT) or a Guide-it^TM^ Mutation Detection Kit (TaKaRa) according to the manufacturer’s instructions, and analyzed by agarose-gel electrophoresis or microchip electrophoresis using MultiNA (SHIMADZU). In PCR-RFLP, the amplified 300–500 bp region for the *SlIAA9*-gRNA2 was digested with AccI (NEB) for 3 h and analyzed by microchip electrophoresis. In sequence analysis, PCR fragments were extracted from the agarose-gel using Wizard^®^ SV Gel and PCR Clean-Up System (Promega) and sub-cloned using a Zero Blunt^TM^ TOPO^TM^ PCR Cloning Kit (Thermo Fisher Scientific). Sanger sequencing of each of the cloned DNAs was performed using a sequencing service (Eurofins Genomics). All primers used for PCR are listed in Supplementary Table S1.

## Results

### Construction of Multiplex CRISPR/Cas9 Vectors

**FIGURE 1:**
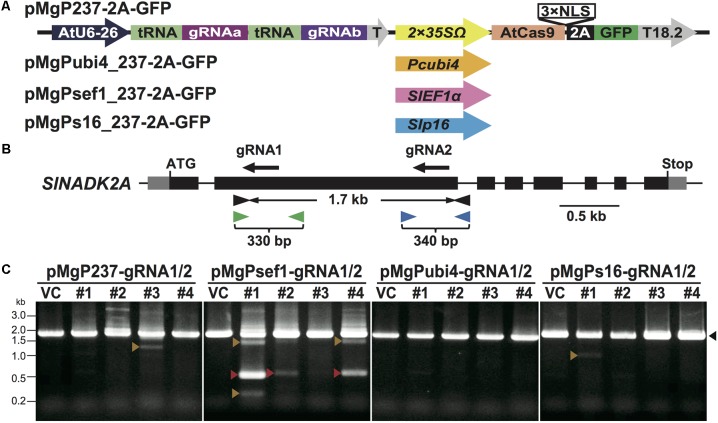
Mutation analyses in the *SlNADK2A* gene of tomato calli using PCR and Cel-1 assay. **(A)** Schematic illustrating multiplex CRISPR/Cas9 vectors using different *Cas9* promoters. AtU6-26: *AtU6 snRNA-26* promoter, T: *AtU6-26* terminator, 2 × 35SΩ: 2 × *CaMV35S* promoter with the omega enhancer sequence, Pcubi4: parsley *Ubiqutin4-2* promoter, SlEF1α: *SlEF1*α promoter, Slp16: *Slp16* promoter, AtCas9: Arabidopsis-codon optimized SpCas9, 2 A: 2 A self-cleavage peptide, NLS: nuclear localization signal, T18.2: Arabidopsis *hsp18.2* terminator. **(B)** The gRNA target sites and primer sites in the *SlNADK2A* gene. The gRNA sites are shown as black arrows. Black, green, or blue arrowheads present primers used to amplify the 1.7 kb fragments including *SlNADK2A*-gRNA1 and *SlNADK2A*-gRNA2, the 330 bp fragments including *SlNADK2A*-gRNA1, or the 340 bp fragments including *SlNADK2A*-gRNA2, respectively. **(C)** Detection of deletion mutations between the target sites by PCR analysis. Black arrowheads; 1.7 kbp WT-sized bands, red arrowheads; deleted PCR fragments (0.5 kbp) of precisely the number of nucleotides between the two gRNAs, orange arrowheads; other size-deleted PCR fragments. Lane numbers indicate tomato callus lines.

Multiplex CRISPR/Cas9 vectors utilizing endogenous tRNA processing enzymes were constructed by modification of the pEgP237-2A-GFP vector (Ueta et al., 2017). Xie et al. (2015) reported that a tRNA–gRNA unit increased the gRNA expression level in rice cells and the mutation frequency in rice target loci. We utilized Arabidopsis codon-optimized Cas9 with a 3× NLS fused to GFP via the self-cleaving 2A peptide in pEgP237-2A-GFP (Ueta et al., 2017), and multiple tRNA–gRNA units were introduced into the vector to express the multiple gRNAs effectively in tomato cells. This newly constructed vector series was named pMgP237-2A-GFP (**Figure 1A**). Using this vector backbone, four different types of promoters were used for *Cas9* (*AtCas9*) expression: *CaMV35S* (pMgP237-2A-GFP), *Pcubi4* (pMgPubi4_237-2A-GFP), *SlEF1*α (pMgPsef1_237-2A-GFP), and *Slp16* (pMgPs16_237-2A-GFP). Previous reports have shown that use of the *Pcubi4* promoter in CRISPR/Cas9 leads to constitutive expression in plant cells (Steinert et al., 2015; Ueta et al., 2017). The *SlEF1*α gene is expressed in meristematic cells such as the root tip and the shoot apical meristem (Pokalsky et al., 1989). The *Slp16* gene is an Arabidopsis ribosomal protein gene that is expressed in the egg cell and ubiquitously in other tissues (Forner et al., 2015; Tsutsui and Higashiyama, 2016).

To evaluate somatic mutations using multiplex CRISPR/Cas9 vectors, two gRNAs, *SlNADK2A*-gRNA1, and *SlNADK2A*-gRNA2 (**Figure 1B**), were first designed to introduce mutations into the tomato *NAD Kinase 2A* gene (*SlNADK2A*; *Solyc06g060060*)—a tomato homolog of the Arabidopsis *NADK2* gene—using the gRNA design “focas” (Osakabe et al., 2016), a web-tool based on the CasOT algorithm to search for potential off-target sites (Xiao et al., 2014) and to evaluate on-target activity (Doench et al., 2014). To assess the efficiency of deletion mutations induced by multiplex genome editing in CRISPR/Cas9, the *SlNADK2A*-gRNA1 and *SlNADK2A*-gRNA2 (named *SlNADK2A*-gRNA1/2) were inserted into the four types of vector. The resulting vectors were transformed into tomato cotyledons by *Agrobacterium*-mediated transformation, and transgenic calli were selected by GFP fluorescence after 4 weeks of culture.

### Detection of Target Mutagenesis in the *SlNADK2A* Gene

PCR analysis was then performed to evaluate mutagenesis mediated by *SlNADK2A*-gRNA1/2 using the multiplex CRISPR/Cas9 vectors, especially if small fragments generated by deletion mutations were detected in the target loci (**Figure 1C**). To evaluate individual mutations at each target site, *SlNADK2A*-gRNA1 or *SlNADK2A*-gRNA2, Cel-1 assays, and PCR analysis were performed using the same selected samples of GFP-positive transgenic calli. In the Cel-1 assay (Supplementary Figure S1), digested bands were detected at each target site in transgenic lines derived from all vectors except pMgPubi4-gRNA2. For the *SlNADK2A*-gRNA1-target site, the mutation rates associated with the different promoters were as follows: *CaMV35S* 38%, *Pcubi4* 14%, *SlEF1*α 26%, and *Slp16* 18% (**Table 1**). Mutations were also detected at the *SlNADK2A*-gRNA2-target site using three vectors; the mutation rates were 38% with the *CaMV35S* promoter, 7% with the *SlEF1*α promoter and 14% with the *Slp16* promoter, whereas the rate was quite low when the *Pcubi4* promoter was used (**Table 1**). When the *SlEF1*α promoter was used for *Cas9* expression, deletion fragments were detected in PCR analysis (33%; 9 mutants with large deletions in 27 transgenic calli) (**Figure 1C**), whereas this type of mutation was not generated frequently by the other three promoters (**Figure 1C**). The results confirm the higher rate of deletion mutations when using the *SlEF1*α promoter compared with the *CaMV35S* promoter.

**Table 1:** Mutation frequencies in *SlNADK2A.*

Vector	gRNA1^∗^		gRNA2^∗^		Deletion^∗∗^		Mutation frequency	
pMgP237-2A-GFP	38%	(5/13)	38%	(5/13)	0%	(0/13)	46%	(6/13)^∗∗∗^
pMgPubi4_237-2A-GFP	14%	(1/7)	0%	(0/7)	0%	(0/7)	14%	(1/7)
pMgPsef1_237-2A-GFP	26%	(7/27)	7%	(2/27)	30%	(8/27)	33%	(9/27)
pMgPs16_237-2A-GFP	18%	(4/22)	14%	(3/22)	0%	(0/22)	32%	(7/22)

^∗^*mutation rates at the target sites by Cel-1 assay. ^∗∗^deletion mutation rates by PCR analysis without gRNA1 to gRNA2 nucleotides. ^∗∗∗^(# mutant calli / # transformant calli)*.

**FIGURE 2:**
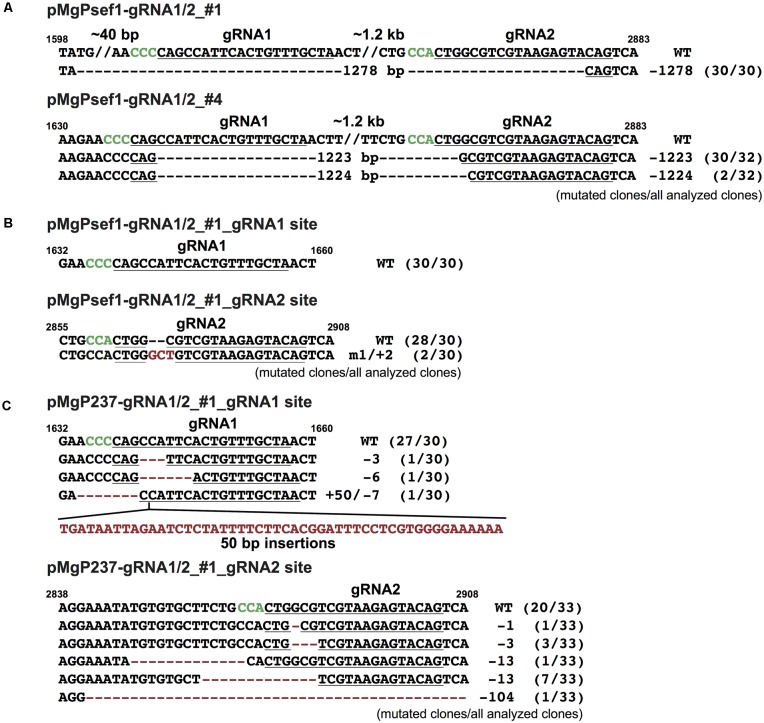
Mutated sequences in transgenic calli harboring pMgPsef1_*SlNADK2A*-gRNA1/2 or pMgP237_*SlNADK2A*-gRNA1/2. **(A)** DNA sequences of the large deleted regions in pMgPsef1_*SlNADK2A*-gRNA1/2 transformed tomato lines #1 and #4. **(B)** DNA sequences of the non-deleted region (**Figure 1C**; WT-sized band) in pMgPsef1_*SlNADK2A-gRNA1/2* transformed tomato line #1. **(C)** DNA sequences of the non-deleted region (**Figure 1C**; WT-sized band) in pMgP237_*SlNADK2A*- gRNA1/2 transformed tomato line #1. gRNA sites are underlined and PAM sequences are in green. The wild-type sequences are shown on top (WT). Except for large deletions, deletions and substitutions are presented as red characters. //; the abbreviation of intermediate sequences.

Approximately 0.5 kbp fragment as the deletion between two gRNA target sites that were identified in the PCR analysis of pMgPsef1_gRNA1/2 lines #1 and #4 (**Figure 1C**, red arrowheads) were then sub-cloned, and the DNA sequences were analyzed. A 1278 bp deletion was detected in the pMgPsef1_gRNA1/2 line #1 and 1223 or 1224 bp deletions were detected in line #4 with low-level mosaicism (**Figure 2**). Furthermore, the sequence data showed that deletion mutations were without intermediate sequences, and that any insertions or substitutions were induced in these lines. Large fragments detected in the PCR analysis of pMgP237_gRNA1/2 line #1 and pMgPsef1_gRNA1/2 line #1 (**Figure 1C**, black arrowheads) were also sub-cloned and subjected to sequence analysis. Various types of mutation were detected at both target sites in pMgP237_gRNA1/2 line #1, but not in pMgPsef1_gRNA1/2 line #1 (**Figures 2B,C**).

**FIGURE 3:**
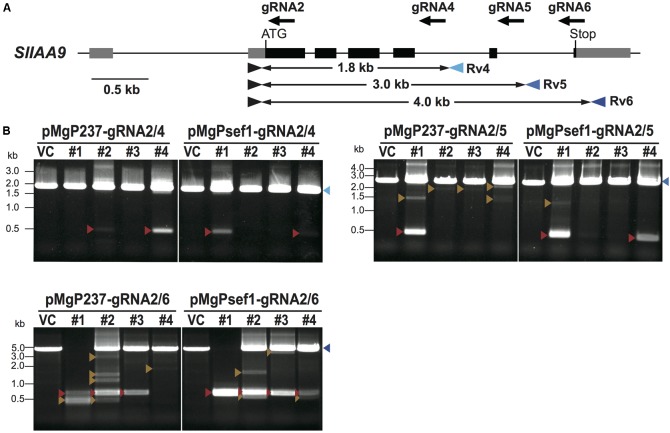
Detection of deletion mutations between the target sites by PCR analysis in *SlIAA9*. **(A)** The gRNA target sites and primer sites in the *SlIAA9* gene. The gRNA sites are shown as black arrows. Blue arrowheads indicate primers used to amplify the 1.8 kb fragments covering the target sites of *SlIAA9*-gRNA2 and *SlIAA9*-gRNA4, the 3.0 kb fragments including *SlIAA9*-gRNA2 and *SlIAA9*-gRNA5, or the 4.0 kb fragments including *SlIAA9*-gRNA2 and *SlIAA9*-gRNA6, respectively. **(B)** Detection of deletion mutations between target sites by PCR analysis. Blue arrowheads; WT-sized bands, red arrowheads; large-deleted fragments of the precise number of nucleotides between two gRNAs, orange arrowheads; other size deletion fragments. VC; vector control tomato plants harboring the empty vector. Lane numbers indicate the tomato callus lines.

When using *SlNADK2A*-gRNA1/2, deletion mutations were detected more frequently when using the *SlEF1*α promoter for *Cas9* expression. To evaluate if *Cas9* expression promoters, especially *SlEF1*α and *CaMV35S* promoters, affect mutation patterns, three gRNAs, *SlIAA9*-gRNA4, *SlIAA9*-gRNA5, and *SlIAA9*-gRNA6, were designed to introduce mutations in the tomato *IAA9* gene (*SlIAA9*) (**Figure 3A**). The *SlIAA9* gene is involved in tomato fruit development, repressing fruit initiation without fertilization, and the *sliaa9* knockout tomato plants generated by CRISPR/Cas9 editing using a single gRNA, *SlIAA9*-gRNA2, showed abnormal leaf shape, and parthenocarpy (Ueta et al., 2017). It has also been shown that the *SlIAA9*-gRNA2 can induce highly efficient mutagenesis in *SlIAA9* to generate knockout tomato lines in the T0 generation (Ueta et al., 2017). The three tRNA–gRNA units, *SlIAA9*-gRNA2/4, *SlIAA9*-gRNA2/5, and *SlIAA9*-gRNA2/6, were inserted into pMgPsef1_237-2A-GFP or pMgP237-2A-GFP, respectively.

**FIGURE 4:**
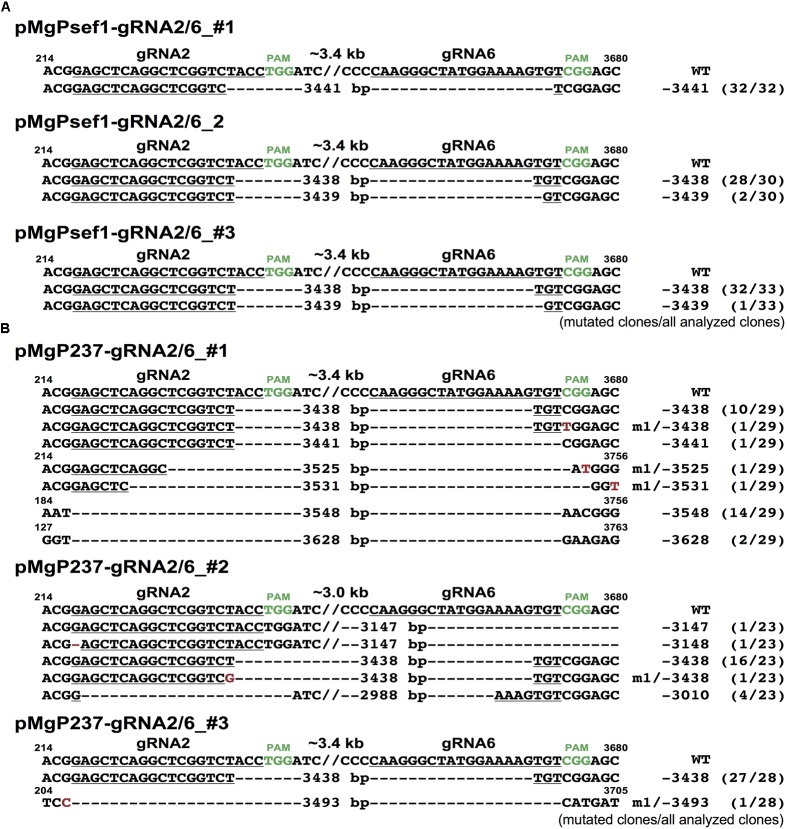
Mutated sequences in transgenic calli harboring pMgPsef1_*SlIAA9*-gRNA2/6 or pMgP237_*SlIAA9*-gRNA2/6 vectors. **(A)** DNA sequences of the large-deleted regions in pMgPsef1_*SlIAA9*-gRNA2/6 transformed tomato lines #1, #2, and #3. **(B)** DNA sequences of the large-deleted regions in pMgP237_*SlIAA9*-gRNA2/6 transformed tomato lines #1, #2, and #3. gRNA targets are underlined and PAM sequences are in green. The wild-type sequences are shown on top (WT). Except for the large deletions, deletions and substitutions are presented as red characters. //; the abbreviation of intermediate sequences.

To investigate deletion mutations at the target sites of *SlIAA9*, PCR analysis was performed on selected GFP-positive transgenic callus. **Figure 3B** shows PCR products from the target sites. Deletion fragments were detected in all six *SlIAA9* vectors, with mutation frequencies as follows: pMgP237_*SlIAA9*-2/4 11%, pMgP237_*SlIAA9*-2/5 27%, pMgP237_*SlIAA9*-2/6 65%, pMgPsef1_*SlIAA9*-2/4 12%, pMgPsef1_*SlIAA9*-2/5 29%, and pMgPsef1_*SlIAA9*-2/6 61% (**Table 2**). The small fragments, which were presumably caused by the precise deletion mutation, were detected in the PCR analysis in pMgPsef1_*SlIAA9*-gRNA2/6 lines #1-#3, and pMgP237-*SlIAA9*-gRNA2/6 lines #1-#3 (**Figure 3B**, red arrowheads) were then sub-cloned and subjected to DNA sequencing. A 3441 bp deletion was detected in pMgPsef1_gRNA2/6 line #1, and 3438 bp and 3439 bp deletions were detected in pMgPsef1_gRNA2/6 both lines #2 and #3 (**Figure 4**) with low-level mosaicism. Furthermore, these sequences exhibited deletion mutations without intermediate sequences, insertions or substitutions. Although the mutation sequences did not vary in pMgP237_*SlIAA9*-gRNA2/6 line #3; various types of large deletion mutations were detected in pMgP237_*SlIAA9*-gRNA2/6 lines #1 and #2 (**Figure 4B**). Together with these results, we concluded that, although expressing *Cas9* from either the *SlEF1*α and *CaMV35S* promoter could generate large deletion mutations, using the *SlEF1*α promoter effectively induced less complex mutation patterns, producing large deletion mutations in which the target sites in between two gRNAs are deleted at the putative cleavage sites.

**Table 2:** Deletion mutations rates in *SlIAA9* without intermediate sequences.

Vector	gRNA2/4		gRNA2/5		gRNA2/6	
pMgP237-2A-GFP	11%	(2/19)^∗^	27%	(6/22)	65%	(17/26)
pMgPsef1_237-2A-GFP	12%	(2/17)	29%	(9/31)	61%	(19/31)

^∗^*(Mutant calli number / transformant calli number)*.

### Tissue-Specific Expression of *SlEF1*α and *CaMV35S* Promoters in Tomato Callus

**FIGURE 5:**
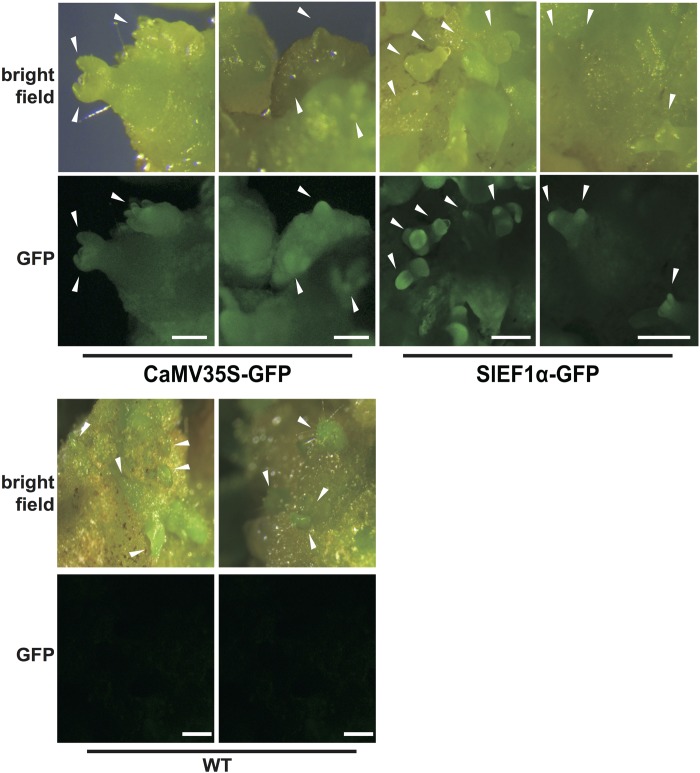
Expression patterns of the *35S* and *SlEF1*α promoters in tomato calli. The transgenic calli harboring the promoter-GFP constructs for *CaMV35S* and *SlEF1*α promoters (3 weeks after transformation). Images of WT calli under the same conditions are also shown. White arrowheads indicate the developing shoot buds. Bar = 500 μm.

We speculated that the promoter expression pattern might control the observed differences in mutation in tomato. To investigate expression patterns using *CaMV35S* and *SlEF1*α promoters in tomato calli, promoter-GFP vectors were constructed and introduced into tomato plants. Strong GFP fluorescence in transgenic tomato harboring the *SlEF1*α-GFP was detected in developing shoot buds in calli 3 weeks after transformation (**Figure 5**). In contrast, GFP expression driven by the *CaMV35S* promoter was detected ubiquitously in the calli, and did not show any tissue specificity (**Figure 5**). These results suggest that by using *CaMV35S* and *SlEF1*α promoters in multiplex CRISPR/Cas9, the variation in *Cas9* expression patterns in the transgenic tomato calli might affect mutation patterns.

## Discussion

Multiplex genome editing systems exploiting CRISPR/Cas9 technology have been developed using different gRNA expression strategies, including systems based on expression of individual and multiple gRNAs, ribozymes, bacterial Csy4 ribonuclease, or tRNA processing enzymes (Li et al., 2013; Xie et al., 2015; Qi et al., 2016; Tang et al., 2016; Čermák et al., 2017; Mercx et al., 2017). Mutation frequency varies with the *Cas9* expression promoter used in the CRISPR/Cas9 system (Gao et al., 2015; Hyun et al., 2015; Wang et al., 2015; Yan et al., 2015; Mao et al., 2016; Osakabe et al., 2016; Tsutsui and Higashiyama, 2016; Osakabe and Osakabe, 2017). In this study, we evaluated targeted mutagenesis using four different *Cas9* expression promoters in multiplex CRISPR/Cas9 based on tRNA processing as a gRNA expression strategy. When using the *SlEF1*α promoter for *Cas9* expression, deletion mutations, in which the target sites in between two gRNAs were deleted at the putative cleavage sites, were induced efficiently in the tomato genome. Furthermore, few mosaic mutations were detected in the target sites compared with similar experiments expressing *Cas9* from the *CaMV35S* promoter, and deletion mutations were yielded by ligation of two Cas9-predicted cut sites without any insertions or substitutions. We also tested closely adjacent targets sites (*SlIAA9*-gRNA2/3, Supplementary Figure S2), and the results showed that the efficiency of multiplex mutagenesis was decreased significantly, despite employing the same highly efficient gRNAs used in simplex genome editing and an efficient tissue-specific promoter, the *SlEF1*α promoter, in the multiplex CRISPR/Cas9. This negative effect may be caused by steric hindrance due to Cas9 proteins binding to closely adjacent targets sites.

The *SlEF1*α gene is expressed in meristematic cells, such as the root tip or the shoot apical meristem in tomato (Pokalsky et al., 1989). Furthermore, heterogeneous expression analysis in tobacco plants has revealed that the *SlEF1*α gene is expressed not only in these latter meristematic tissues but also in germ cells (Ursin et al., 1991). We also observed high GFP fluorescence in shoot buds developing from calli harboring the *SlEF1*α promoter-GFP vector (**Figure 5**). The *SlEF1*α gene is homologous to the *AtEF1*α gene. The expression level of the *AtEF1*α promoter is approximately twofold higher than that of the *CaMV35S* promoter in Arabidopsis protoplast transient expression and it has high activity in meristematic cells in Arabidopsis plants (Axelos et al., 1989; Osakabe et al., 2016). Furthermore, *Cas9* expression from the *AtEF1*α promoter had been shown to induce mutagenesis effectively in the Arabidopsis genome (Osakabe et al., 2016) and the present study has revealed that *Cas9* expressed strongly in the early stages of shoot formation can effectively induce mutations with deletion of the nucleotides in between two gRNAs in the tomato genome. Using the *SlEF1*α promoter for *Cas9* expression, mosaic mutations were clearly decreased in the tomato genome, whereas using constitutive promoters for *Cas9* expression induces several types of mutations in various cells simultaneously or at different stages during plant tissue culture. Although further analysis to determine the precise plant stages of *SlEF1*α promoter expression is needed to elucidate the specific mechanisms of mutation, CRISPR/Cas9 system-induced mutations at specific stages with a tissue-specific promoter in early differentiation stages decreases the mosaic mutation rate and enhances deletion mutations without any insertion or substitutions when the gRNAs pair.

Previous studies on multiplex genome editing have shown that large deletion mutations in target sites were detected with various types of indels at the target sites (Xie et al., 2015; Čermák et al., 2017). By using the *SlEF1*α promoter for *Cas9* expression in the present study, the deletion mutations were ligated between the target sites without any insertions or substitutions, suggesting specific DNA repair mechanisms. There are three major pathways of DNA repair: NHEJ, microhomology-mediated end joining (MMEJ), and homologous recombination (HR) (Rodgers and McVey, 2016). The HR pathway has been used in gene targeting; however, the efficiency is quite low in plants (Li et al., 2013, 2016). Since deletion mutations were detected with highly efficient by using the *SlEF1*α promoter for *Cas9* expression in this study, this system could be used for efficient gene targeting, for example, in the PITCh system, in which an MMEJ-assisted gene knock-in can be used for genome editing in mammalian cells (Sakuma et al., 2016), because the multiplex CRISPR/Cas9 system coupled with the *SlEF1*α promoter could maintain two microhomology sequences at the Cas9-cut sites without any deletion. This type of mutation could also be useful to create the precise deletion required in, for example, domain analysis. We also focus on the low mosaicism in the T0 generations when using the *SlEF1*α, which can be utilized effectively to isolate homozygotes in the T1 generation. Multiplex genome editing using a tRNA processing system with a tissue-specific expression promoter directing *Cas9* expression can be utilized to induce multiple target mutagenesis. This result suggests that further applications of multiplex genome editing can be expected when tissue- or temporal-specific promoters are selected in plant tissue culture. Multiplex genome editing is a useful technique for various purposes, such as the deletion of a target domain sequence or multiple target mutagenesis. Optimizing the CRISPR/Cas9 system, especially the choice of *Cas9* expression promoter for multiplex genome editing, would further contribute to basic molecular studies and molecular breeding techniques in various plant species, including useful crops, as one of the most useful genome editing tools in plant genome engineering.

## Author Contributions

RH performed most of the research, analyzed the data, and wrote the manuscript. RU and CA produced the CRISPR/Cas9 transgenic lines. YO supervised the research and wrote the manuscript. KO designed, led, and coordinated the overall study.

## Conflict of Interest Statement

The authors declare that the research was conducted in the absence of any commercial or financial relationships that could be construed as a potential conflict of interest.
