# Supplementary material for: Efficient Multiplex Genome Editing Induces Precise, and Self-Ligated Type Mutations in Tomato Plants
Source: Front Plant Sci. 2018 Jul 3;9:916. doi: 10.3389/fpls.2018.00916 (PMC6037947; doi:10.3389/fpls.2018.00916)
Supplement: Supplementary file 1 [file Table_1.pdf]

Supplemental Table 1. Primer lists for construction of multiplex CRISPR/Cas9 vectors and mutation analyses.

Fw_Psef1 cloning	TTCCGCGGTGGCGCGCCGTAGAGTTCATTTAAAATTCTT	For Constructing the pMgPsef1_237-2A-GFP
Rv_Psef1 cloning	GTAAAAATACTCTAGACACTAAGAAACTGCATTTACA	For Constructing the pMgPsef1_237-2A-GFP
Fw_Ps16 cloning	TTCCGCGGTGGCGCGCCACATGTTCCAAACCTAA	For Constructing the pMgPs16_237-2A-GFP
Rv_Ps16 cloning	GTAAAAATACTCTAGCTGAATCGGCTCTCGATGAA	For Constructing the pMgPs16_237-2A-GFP
Fw_SlNADK2A_tgRNA-gRNA1	TTGGGTCTCCAAACCAGCCATTCACTGTTTGCTACTGCACCAGCCGGGAATCGAA	For amplifying the tRNA-gRNA unit of SlNADK2A_gRNA1/2
Rv_SlNADK2A_tgRNA-gRNA2	TTGGGTCTCGTGCAGCTGTACTCTTACGACGCCAGGTTTTAGAGCTAGAAATAGCA	For amplifying the tRNA-gRNA unit of SlNADK2A_gRNA1/2
Fw_SlIAA9_tgRNA-gRNA2	TTGGGTCTCGTGCAGAGCTCAGGCTCGGTCTACCGTTTTAGAGCTAGAAATAGCA	For amplifying the tRNA-gRNA units of SlIAA9_gRNA2/3, SlIAA9_gRNA2/4, SlIAA9_gRNA2/5, or SlIAA9_gRNA2/6
Rv_SlIAA9_tgRNA-gRNA3	TTGGGTCTCCAAACCACCTCTTTCGGGAGACTGACTGCACCAGCCGGGAATCGAA	For amplifying the tRNA-gRNA unit of SlIAA9_gRNA2/3
Rv_SlIAA9_tgRNA-gRNA4	TTGGGTCTCCAAACACCTGAACTCTTACTGCATGCTGCACCAGCCGGGAATCGAA	For amplifying the tRNA-gRNA unit of SlIAA9_gRNA2/4
Rv_SlIAA9_tgRNA-gRNA5	TTGGGTCTCCAAACCCCGGAAGATCACTAGTATTCTGCACCAGCCGGGAATCGAA	For amplifying the tRNA-gRNA unit of the SlIAA9_gRNA2/5
Rv_SlIAA9_tgRNA-gRNA6	TTGGGTCTCCAAACACACTTTTCCATAGCCCTTGCTGCACCAGCCGGGAATCGAA	For amplifying the tRNA-gRNA unit of the SlIAA9_gRNA2/6
Fw_Psef1	GCCAGTGCCAAGCTTGCGCCGTAGAGTTCATTTAAAA	For constructing the pRI SlEF1α-GFP
Rv_Psef1	TGTGATGTATCTAGACACTAAGAAACTGCATTTACA	For constructing the pRI SlEF1α-GFP
Fw_SlNADK2-gRNA1	TATTTTCAGAGGTGAAATGAAGAGG	For amplifying SlNADK2A-gRNA1/2 region or Cel-1 assay of SlNADK2A-gRNA1 region
Rv_SlNADK2-gRNA1	GCAGACTGAGTCTCTTCTTTTGATGTTGA	For Cel-1 assay of SlNADK2A-gRNA1 region
Fw_SlNADK2-gRNA2	CATCACACCTTCACTGGAAGATAACTTG	For Cel-1 assay of SlNADK2A-gRNA2 region
Rv_SlNADK2-gRNA2	CAGCAGTAGTAAATGAAAATCACAAGGTAA	For amplifying SlNADK2A-gRNA1/2 region or Cel-1 assay of SlNADK2A-gRNA2 region
Fw_SlIAA9_gRNA2	GGAGGAGGAGGGCCAGAGTAATGTAA	For amplifying SlIAA9-gRNA2/4, SlIAA9-gRNA2/5, or SlIAA9- gRNA2/6 region and mutation analyses of SlIAA9-gRNA2/3 region
Rv_SlIAA9_gRNA3	GTTGCCACTAACTACTGTTTTCTGCGAT	For mutation analyses of SlIAA9-gRNA2/3 region
Rv_SlIAA9_gRNA4	GAAGGCAGCATGAATCTTGA	For amplifying SlIAA9-gRNA2/4 region
Rv_SlIAA9_gRNA5	AACTTTCATGACGAACAGCTG	For amplifying SlIAA9-gRNA2/5 region
Rv_SlIAA9_gRNA6	CTTAATCAAACGACAAACGTTGGGTC	For amplifying SlIAA9-gRNA2/6 region
