# Supplementary material for: Efficient Multiplex Genome Editing Induces Precise, and Self-Ligated Type Mutations in Tomato Plants
Source: Front Plant Sci. 2018 Jul 3;9:916. doi: 10.3389/fpls.2018.00916 (PMC6037947; doi:10.3389/fpls.2018.00916)
Supplement: Supplementary file 2 [file Data_Sheet_1.PDF]

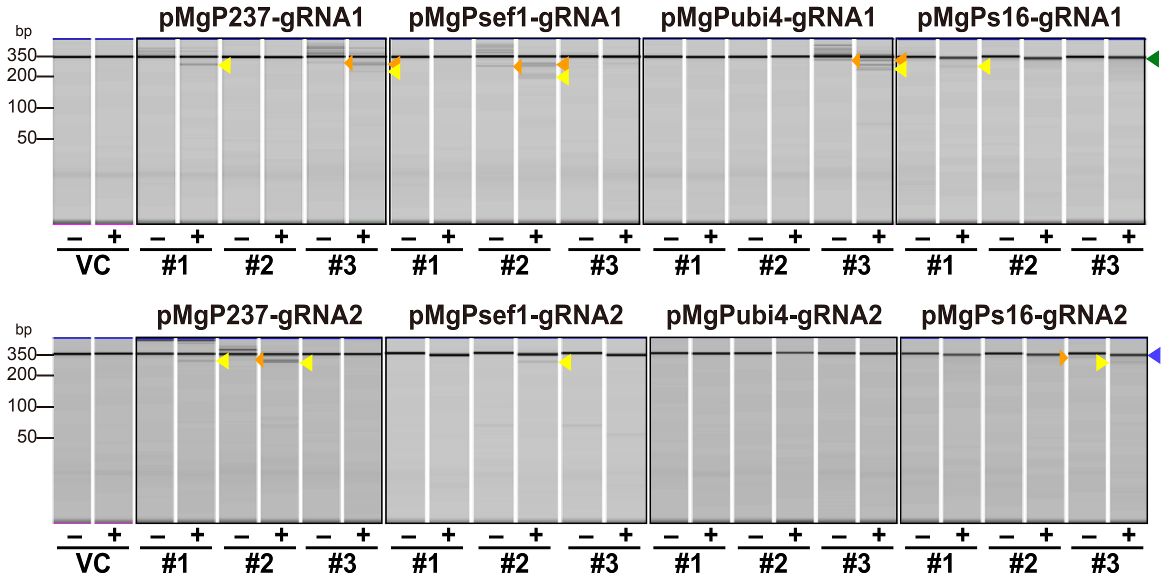

Supplementary Fig. 1. Mutation analysis of *SlNADK2A*-gRNA1 and *SlNADK2A*-gRNA2 sites by Cel-1 assay.

Mutation analysis was performed by the microchip electrophoresis using MultiNA to detect digested fragments higher sensitivity than agarose-gel electrophoresis. The gel image was obtained by MultiNA software based on peak charts shows detection of mutations by Cel-1 assay at the target site of each vectors. Green arrowheads indicate the untreated or non-mutation fragments (330 bp), and yellow arrowheads indicate the digested fragments (260 bp) by Cel-1 assay in *SlNADK2A*-gRNA1 sites (upper panel). Blue arrowheads present the untreated or non-mutation fragments (340 bp), and yellow arrowheads indicate the digested fragments (260 bp) by Cel-1 assay in *SlNADK2A*-gRNA2 sites (lower panel). Orange arrowheads; deletion fragments. –; untreated PCR products. +; treated with Cel-1 nuclease. VC; vector control tomato plants harboring the empty vector. The numbers indicate the tomato calli lines.
