# Supplementary material for: Efficient Multiplex Genome Editing Induces Precise, and Self-Ligated Type Mutations in Tomato Plants
Source: Front Plant Sci. 2018 Jul 3;9:916. doi: 10.3389/fpls.2018.00916 (PMC6037947; doi:10.3389/fpls.2018.00916)
Supplement: Supplementary file 3 [file Data_Sheet_2.PDF]

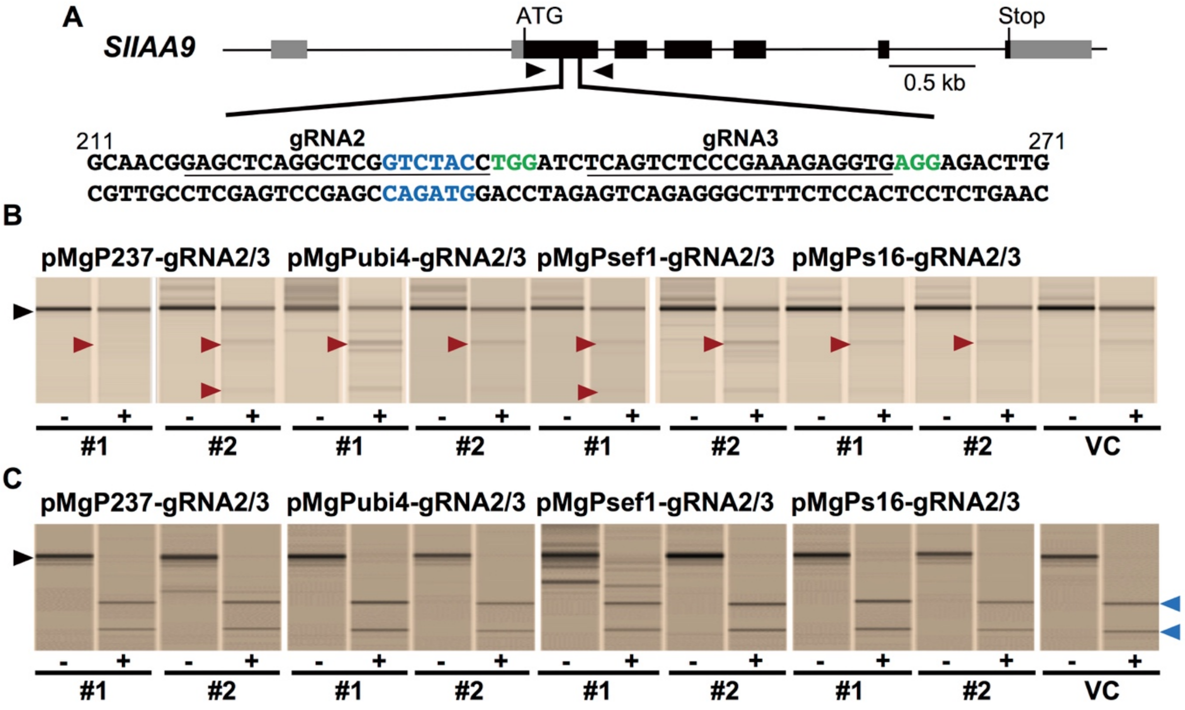

Supplementary Fig. 2. Mutation analyses of *SlIAA9*-gRNA2 and *SlIAA9*-gRNA3 sites by Cel-1 assay and PCR-RFLP

(A) The gRNA target sites and primer sites for *SlIAA9*-gRNA2 and *SlIAA9*-gRNA3 in the *SlIAA9* gene. Black arrowheads present primers used to amplify the 350 bp fragments including *SlIAA9*-gRNA2 and *SlIAA9*-gRNA3 target sites. Blue characters are recognition sites of *Acc*I.

(B) Mutation analysis of *SlIAA9*-gRNA2 and *SlIAA9*-gRNA3 sites by Cel-1 assay. Black arrowheads indicate the untreated or non-mutation fragments (350 bp), and red arrowheads indicate the digested fragments (230 bp and 120 bp) by Cel-1 nuclease.

(C) Mutation analysis of *SlIAA9*-gRNA2 sites by PCR-RFLP. Black arrowheads indicate untreated or mutation fragments (350 bp), and blue arrowheads indicate the digested fragments (210 bp and 140 bp) by *Acc*I in *SlIAA9*-gRNA2 sites. –; untreated PCR products. +; treated with Cel-1 nuclease or *Acc*I. VC; vector control tomato plants harboring the empty vector. The numbers indicate the tomato calli lines.
